# *OsACOS12*, an orthologue of Arabidopsis acyl-CoA synthetase5, plays an important role in pollen exine formation and anther development in rice

**DOI:** 10.1186/s12870-016-0943-9

**Published:** 2016-11-21

**Authors:** Yueling Li, Dandan Li, Zongli Guo, Qiangsheng Shi, Shuangxi Xiong, Cheng Zhang, Jun Zhu, Zhongnan Yang

**Affiliations:** College of Life and Environment Sciences, Shanghai Normal University, 100 Guilin Road, Shanghai, 200234 China

**Keywords:** *Oryza sativa*, *OsACOS12*, Male sterility, Pollen exine, Anther cuticle

## Abstract

**Background:**

Sporopollenin is a major component of the pollen exine pattern. In Arabidopsis, acyl-CoA synthetase5 (*ACOS5*) is involved in sporopollenin precursor biosynthesis. In this study, we identified its orthologue, *OsACOS12*, in rice (*Oryza sativa*) and compared the functional conservation of *ACOS* in rice to Arabidopsis.

**Results:**

Sequence analysis showed that OsACOS12 shares 63.9 % amino acid sequence identity with ACOS5. The *osacos12* mutation caused by a pre-mature stop codon in *LOC_Os04g24530* exhibits defective sexine resulting in a male sterile phenotype in rice. In situ hybridization shows that *OsACOS12* is expressed in tapetal cells and microspores at the transcript level. The localization of OsACOS12-GFP demonstrated that OsACOS12 protein is accumulated in tapetal cells and anther locules. *OsACOS12* driven by the *ACOS5* promoter could partially restore the male fertility of the *acos5* mutant in Arabidopsis.

**Conclusions:**

*OsACOS12* is an orthologue of *ACOS5* that is essential for sporopollenin synthesis in rice. *ACOS5* and *OsACOS12* are conserved for pollen wall formation in monocot and dicot species.

**Electronic supplementary material:**

The online version of this article (doi:10.1186/s12870-016-0943-9) contains supplementary material, which is available to authorized users.

## Background

Male reproductive development is an essential biological process for the propagation of flowering plants. Pollen development is the major event of male reproduction. Developmental defects leading to male sterility are widely used for hybrid production in agriculture [1]. During pollen development, pollen wall formation is a key process required for pollen viability and male fertility. The pollen wall structure divides into the outer exine and the inner intine. The exine is further divided into a species-specific sexine and a flat nexine [2]. The major composition of the sexine is sporopollenin [3], while the nexine is mainly composed of glycoproteins [4]. The biological function of the sexine layer is to provide an external barrier for adapting the terrestrial environment to ensure microgamete survival in land plants to resist various environmental stresses and microbial attacks [5, 6]. The sexine patterning also acts as an important feature of plant taxonomic classifications [7].

Sporopollenin, the major constituent of the sexine, was considered to be a complex polymer primarily composed of long-chain fatty acids, oxygenated aromatic rings and phenylpropanoic acids [8]. The tapetal layer is an essential tissue required for normal sexine development and pollen maturity [9]. Based on cytological and molecular evidence, the material of sporopollenin precursors originate from tapetal cells [10, 11]. The sporopollenin precursors are initially deposited at the mould of the sexine to form probaculae and protectum structures. After a microspore is released, the exine structure increases in size with continuous deposits and polymerization of sporopollenin until the decorated sexine pattern is formed [12]. However, the exact composition of sporopollenin precursors is not clear. In Arabidopsis, several genes have been reported to be involved in the complex biochemical pathways of sporopollenin precursor formation, including CYP703A2, CYP704B1 and MS2 for fatty-acid-derived compound metabolism, PKSA/B and TKPR1/2 for phenylpropanoids synthesis, and ABCG26 for transportation [13–19]. All these genes are expressed in the tapetal layer at the transcript level. On the protein level, MS2 is localized in tapetal cells, while CYP703A2 is in both tapetal cells and the anther locule [19, 20]. It is likely that the last several steps of sporopollenin precursor synthesis occur in the locule.

The sporopollenin is a general constituent that has been widely found in moss, ferns, gymnosperms and angiosperms. Sporopollenin synthesis seems to share common metabolic pathways in various species. The tapetum directly provides materials for pollen wall formation. The genetic pathway for tapetum development is generally conserved between rice and Arabidopsis [21]. In rice, several sporopollenin enzymes have been identified [22–25]. The biological functions of these enzymes and the metabolic pathways for sporopollenin synthesis were very conserved between rice and Arabidopsis. However, the anther cuticle had defects in mutations of the rice genes discussed above, whereas homologous mutants in Arabidopsis did not show obvious morphological changes in the anther walls. It was suggested that the lipidic pathway was diversified in rice [22, 25].

*ACOS5* encodes a fatty acyl-CoA synthetase (ACOS) for sporopollenin precursor synthesis in Arabidopsis [26, 27]. There are nine fatty acyl-CoA synthetase encoding genes in rice [28]. In this study, we characterized an orthologue of Arabidopsis *ACOS5* in rice, *OsACOS12* (*LOC_Os04g24530*). A knockout of this gene led to a male sterile phenotype in rice with a defective sexine layer and anther cuticle. The tapetal and anther locule localization of the OsACOS12 protein suggested that there was synchronous biosynthesis and transportation of sporopollenin precursors. The expression of the *OsACOS12* gene in the Arabidopsis *acos5* mutant partially restored the male sterile phenotype, which indicated that the acyl-CoA synthetase gene is a conserved function between Arabidopsis and rice.

## Results

### *OsACOS12* in *O. sativa* is an orthologue of *ACOS5* in *A. thaliana*

**Fig. 1:**
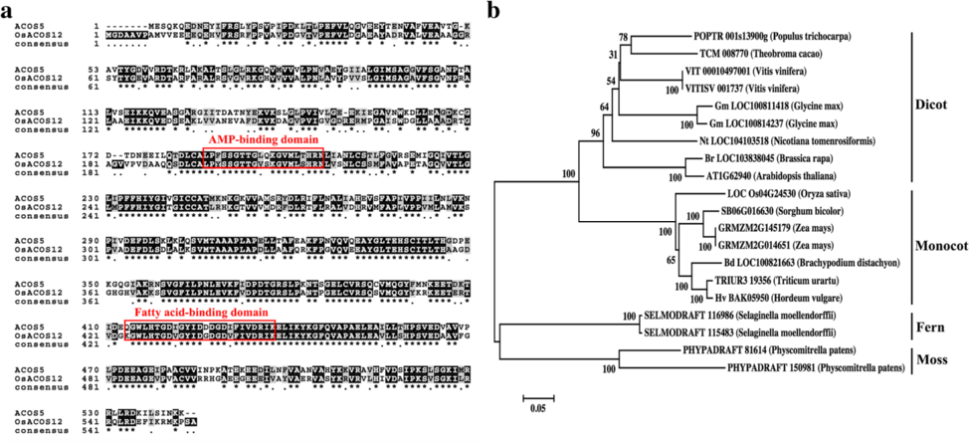
*OsACOS12* in *O. sativa* is an orthologue of *ACOS5* in Arabidopsis. **a** Amino acid sequences alignment of *OsACOS12* and *ACOS5*. The sequences were aligned using Clustal W and displayed using BOXSHADE. **b** A neighbour-joining phylogenetic tree of *OsACOS12* and its orthologues in different species. Bootstrap values are the percentage of 1,000 replicates. The conserved AMP binding domain and fatty acid binding domain are indicated

BLASTP analysis using Arabidopsis ACOS5 protein sequence yield the *LOC_Os04g24530* in rice encoding a fatty acyl-CoA synthetase (http://rice.plantbiology.msu.edu/). The sequence of LOC_Os04g24530 also showed the highest sequence similarity with ACOS5 in Arabidopsis genome (63.9 % amino acid sequence identity, Fig. 1a). *LOC_Os04g24530* was designated *OsACOS12* previously [26]. The large superfamily of acyl-activating enzymes contains putative motifs for AMP-binding and fatty acid-binding [29, 30]. These motifs are highly conserved between *OsACOS12* and *ACOS5* (Fig. 1a). The homologues of OsACOS12 protein have been identified in various plant species by a BLASTP search according to the GenBank database. No orthologue could be identified in the genome of the green alga. Phylogenetic analysis shows OsACOS12 and its homologues formed four distinct clades. The homologues from *Physcomitrella* and *Selaginella* formed two distinct clades that diverged early in land plant evolution. The homologues from dicotyledoneae and monocotyledon species form two other clades (Fig. 1b). Sequence analysis demonstrated that ACOS enzymes are apparently present in land plants, which supports a possible role for them in the biosynthesis of sporopollenin, which is the demand for protecting gametophytes to adapt to a land environment.

### *osacos12* mutant shows complete male sterility

**Fig. 2:**
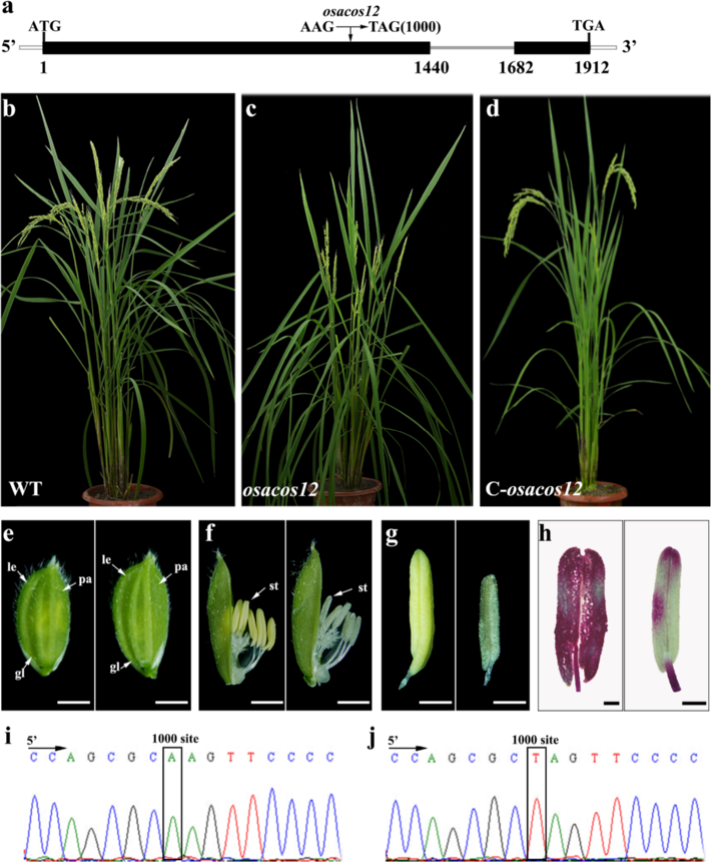
Isolation of the rice *osacos12* mutant with complete male sterility. **a** The gene structure and position of the nucleotide change in *osacos12*. The black boxes indicate exons. **b**-**d** The wild-type (WT) plant, *osacos12* mutant and complementation plant after the heading stage. **e** Comparison of the WT plant (left) and *osacos12* mutant panicles (right) at the heading stage. **f** The spikelets of the WT plant (left) and an *osacos12* mutant (right) after removing the palea. **g** The anther of a WT plant (left) and an *osacos12* mutant (right). **h** Alexander staining of the WT plant (left) and an *osacos12* mutant anther (right). **i** and **j** Identification of the *OsACOS12* gene in a WT plant and *osacos12* mutant by sequencing (position 1000). Bars = 100 μm in **e**-**g** and 200 μm in **h**

To characterize the function of *OsACOS12*, we obtained an allele of *LOC_Os04g24530* using Targeting Induced Local Lesions In Genomes (Tilling) technology from the ethyl methane sulfonate-induced population of rice Zhonghua11 (*O. sativa* ssp. Japonica) [31]. Sequence analysis revealed a point mutation from A to T, in the 1000th base downstream of the start codon of the *LOC_Os04g24530* genomic sequence in the mutant. This transition caused a premature termination (AAG-TAG) at the first exon of *LOC_Os04g24530* (Fig. 2a). The *osacos12* mutant exhibited normal vegetative and spikelet development (Fig. 2c, e). However, the mutant anthers had a white colour without pollen grains inside, which led to complete male sterility (Fig. 2c, f-h). Reciprocal crosses with the wild type indicted that female fertility was not affected in the *osacos12* mutant. The fertile and sterile plants of the F_2_ population segregated with a 3:1 ratio (86:23) indicated that there was a single recessive sporophytic mutation for *osacos12*. To complement the *osacos12* mutant phenotype, the *OsACOS12* genomic fragment fused with GFP driven by its own promoter (1428 bp) was constructed and transformed into heterozygous *OsACOS12* seeds. Of the 32 transgenic lines, 7 were identified to have a homozygous *osacos12* mutant background (Fig. 2i-j). All of these homozygous lines exhibited normal fertility (Fig. 2d). These results demonstrate that *OsACOS12* was responsible for the male fertility of the *osacos12* mutant.

### Pollen sexine formation and anther cuticle are defective in *osacos12*

**Fig. 3:**
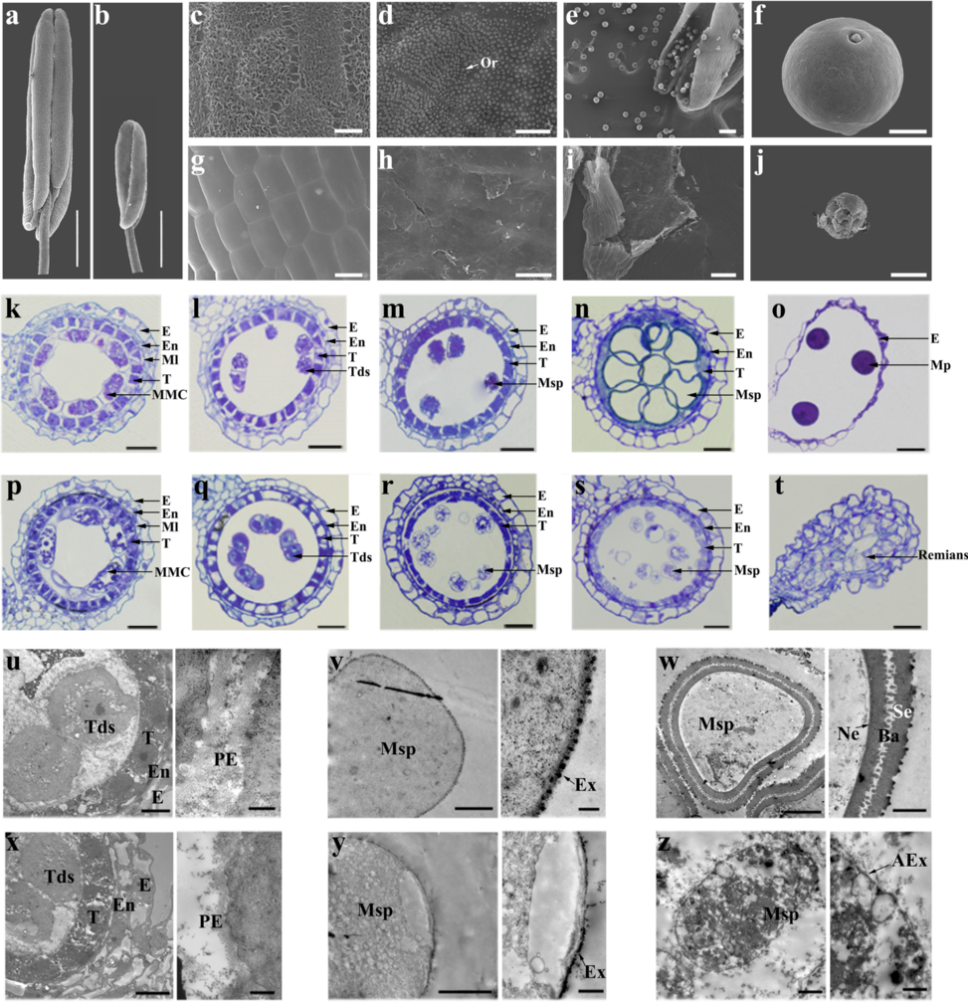
The defective anther cuticle and pollen sexine formation in *osacos12*. **a** and **b** SEM images for the WT and *osacos12* anthers. **c**-**j** SEM observation for the epidermal surface of the WT (**c**) and *osacos12* (**g**) anthers, the inner surface of WT (**d**) and *osacos12* (**h**) anthers, and the pollen grains in WT (**e** and **f**) and *osacos12* (**i** and **j**) anthers. Or, orbicule; Bars = 500 μm in **a** and **b**, 100 μm in **e**, **i**, 10 μm in **c**, **g**, **f**, **j** and 5 μm in **h**, **d. k**-**t** Semi-thin cross-sectional analysis of anther development of WT (**k**-**o**) and the *osacos12* mutant (**p**-**t**) during the anther development stages. E, epidermis; En, endothecium; ML, middle layer; T, tapetum; MMC, microspore mother cell; Tds, tetrads; Msp, microspore. Bars = 20 μm. **u**-**z** TEM observation for WT (**u**-**w**) and *osacos12* (**x**-**z**) pollen development from stages 8–10. The boxed image on the right of each panel was enlarged from the left region. AEX, abnormal exine; Ba, bacula; E, epidermis; En, endothecium; Ex, exine; Msp, microspore; Ne, nexine, PE, primexine; Se, sexine; T, tapetum; Tds, tetrads. Bars = 5 μm and 500 nm in **u**, **x**, 2 μm and 500 nm in **v**, **y**, 5 μm and 1 μm in w, 2 μm and 1 μm in **z**

Scanning electron microscopy (SEM) was used to elucidate the abnormal morphological defects of anther development in the *osacos12* plant. The *osacos12* anther was much shorter and smaller compared to that of the wild type (Fig. 3a-b). In the wild type, the anther surface was covered by cuticle (Fig. 3c), and orbicules were intensively distributed on the inner surface (Fig. 3d). However, *osacos12* anther surface was lack of these materials and looks smooth (Fig. 3g), and orbicules were barely detected in the inner surface (Fig. 3h). The wild type anther was filled with mature pollen grains (Fig. 3e-f). In contrast, only a few remnants of degenerated pollen could be observed in the *osacos12* anther (Fig. 3i-j). In the complementary transgenic lines, the anther surface and pollen exine were restored (Additional file 1: Figure S1).

We subsequently obtained semi-thin transverse sections to understand the detailed defects of pollen development in *osacos12*. There were no detectable differences between wild type and *osacos12* during early anther development. The microspore mother cells (MMCs) and tetrads of *osacos12* appeared to be comparable with the wild type (Fig. 3k-l, p-q). In the wild type, newly released microspores of the wild type were angular in shape (Fig. 3m) and became enlarged and vacuolated (Fig. 3n). In *osacos12* plants, the released microspores contained much less cytoplasm (Fig. 3r) and were degenerated before/during volume enlargement (Fig. 3s). No mature pollen grains were observed in the locule of *osacos12* at later stages of anther development (Fig. 3t). The defective phenotype of *osacos12* was similar to *acos5* in Arabidopsis.

To further clarify the details of the abnormal exine development of *osacos12* pollen, anther samples were investigated using transmission electron microscopy (TEM). During the tetrad stage, primexine is formed between callose wall and plasma membrane. It is critical for pollen wall pattern. The primexine formation in *osacos12* is consistent with that in wild type at tetrad stage (Fig. 3u, x). At stage 9, the pollen exine in *osacos12* was not as deeply stained as that in the wild type, indicating an abnormal sporopollenin deposition (Fig. 3v, y). At stage 10, the exine layer of microspore was formed with gradually deposition of sporopollenin precursors in the wild type (Fig. 3w). However, in *osacos12*, no sporopollenin precursor accumulated on the microspore surface resulted in absent exine layer phenotype of collapsed pollen grains (Fig. 3z). Additionally, there were no obvious aberrations in the appearance of the tapetum in the *osacos12* mutant (Additional file 2: Figure S2). These observations revealed that the exine formation and cuticle structures of anther epidermis were abnormal in *osacos12*.

### Defective wax components in *osacos12* anthers

**Fig. 4:**
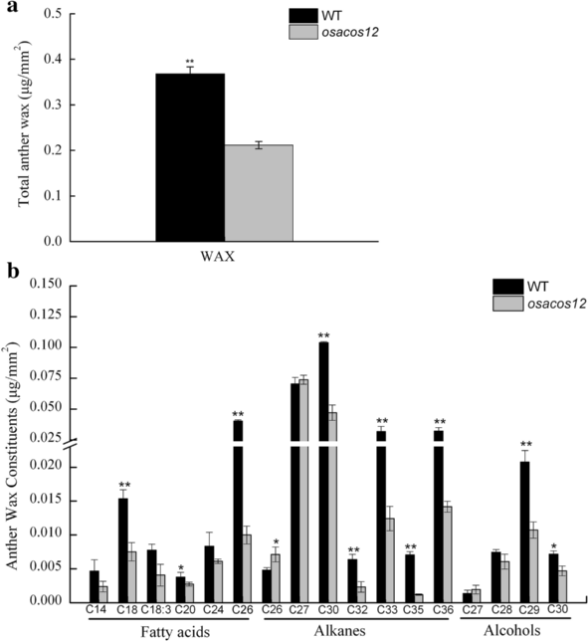
Anther cuticle wax constitutions in WT and *osacos12*. **a** The total amount of anther wax per unit of anther surface area. **b** The amounts of anther wax per unit of anther surface area. Compound names are abbreviated as follows: C14, myristic acid; C18, stearic acid; C18:3, linolenic acid; C20, arachidic acid; C24, lignoceric acid; C26, hexacosanoic acid; C26, hexacosane; C27, heptacosane; C30, triacontane; C32, dotriacontane; C33, tricosane; C35, pentatriacontane; C36, hexatriacontane; C27, 1-heptacosanol; C28, 24-epicampesterol; C29, sitosterol; C30, 1-triacontanol. Values are the mean ± SD (*n* = 3). *, *P* < 0.05; **, *P* < 0.01 (Student’s *t* test)

The defective anther cuticle and sporopollenin in *osacos12* suggested that the lipidic mechanisms were aberrant in the mutant. To confirm this point, we performed gas chromatography–mass spectrometry (GC-MS) to quantify wax extracts from whole anthers of both the wild type and *osacos12* mutants. The results showed that the total cuticular wax amount was reduced by approximately 42.6 % in the mutant (Fig. 4a), which contributed to the significant reduction of most wax constituents. The components of wax, including long-chain fatty acids (C14 to C26), alkanes (C30 to C36) and alcohols (C28 to C30) were significantly decreased in the *osacos12* mutant (Fig. 4b). Therefore, chemical analysis indicated that *OsACOS12* was involved in the synthesis of lipidic compounds during rice anther development.

### OsACOS12 is located in the tapetum and anther locule

**Fig. 5:**
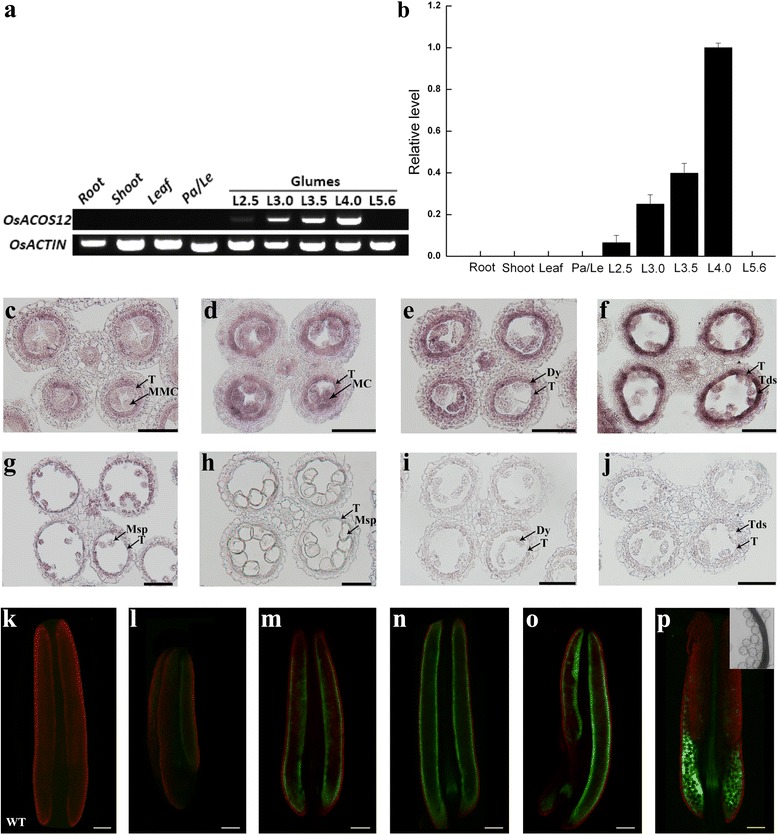
OsACOS12 is specifically expressed in the anther. **a** RT-PCR analysis of RNA isolated from various tissues using *OsACOS12* and *OsACTIN* primer sets. Le, lemma; Pa, palea; L2.5, Glumes length 2.5 mm; L3.0, Glumes length 3.0 mm; L3.5, Glumes length 3.5 mm; L4.0, Glumes length 4.0 mm; L5.6, Glumes length 5.6 mm. **b** Quantitative real-time PCR analysis of *OsACOS12*. The *OsACTIN* gene served as the reference. Data are shown as the mean ± SD (*n* = 3). **c**-**j** In situ hybridization of *OsACOS12* in WT anthers. The anthers at the MMC stage (**c**), early meiosis stage (**d**), tetrad stage (**e** and **f**), microspore release stage (**g**), and microspore vacuolate stage (**h**) hybridized with an *OsACOS12* antisense probe. The anthers at the tetrad stage (**i**-**j**) hybridized with an *OsACOS12* sense probe. Msp, microspore; T, tapetum; Tds, tetrads. MMC, microspore mother cell; MC, meiotic cell; Dy, dyad cell. Bars = 50 μm. **k**-**p** Fluorescence confocal images of the OsACOS12-GFP fusion proteins at different stages. The green channel shows the GFP expression (530 nm), and the red channel shows the chlorophyll autofluorescence (>560 nm). The bright-field images of (**p**) show that these fusion proteins are not localized to the microspores. Bars = 10 μm; 100 μm

In Arabidopsis, *ACOS5* is expressed in tapetal cells and microspores from late stage 5 to stage 8, as shown through in situ hybridization [26]. To analyse the expression of *OsACOS12* in rice, semi-quantitative RT-PCR analysis was performed. *OsACOS12* expression was detected in the anthers with glume lengths of 2.5 mm to 4.0 mm but was not detected in roots, shoots, leaves and palea/lemma (Fig. 5a). This result was further confirmed by quantitative real-time PCR analysis (Fig. 5b). Spatial and temporal expressions of *OsACOS12* were detected by an RNA in situ hybridization analysis using an *OsACOS12*-specific probe. The *OsACOS12* was initially expressed in the tapetal layer and microspore mother cells at the beginning of meiosis (Fig. 5c-d). The signal was increased significantly and reached the highest level during the tetrad stage (Fig. 5e-f). At microspores stage, the signal of *OsACOS12* transcripts was obviously decreased in the tapetum and microspores (Fig. 5g-h). In the control, only background signal was detected using a sense probe at tetrad stage (Fig. 5i-j).

To understand the expression of OsACOS12 at the protein level, we analysed the GFP signal in the complemented transgenic lines (Fig. 2d). In this transgenic line, OsACOS12-GFP can complement the *osacos12* phenotype. Thus, the GFP signal in this line represented the OsACOS12-GFP protein level. The GFP signal was detected within the tapetal cells, which formed a circle in the anther (Fig. 5m-o). In the late stages of anther development, the fluorescence signal transferred into the locule (Fig. 5p). However, the GFP signal was not expressed inside the microspores (Fig. 5p). The wild type anther of rice was used as a negative control (Fig. 5k). These results indicate that OsACOS12 is accumulated in tapetal cells and anther locules according to different anther stages.

### *OsACOS12* could partially fulfil the function of *ACOS5* for pollen development in Arabidopsis

**Fig. 6:**
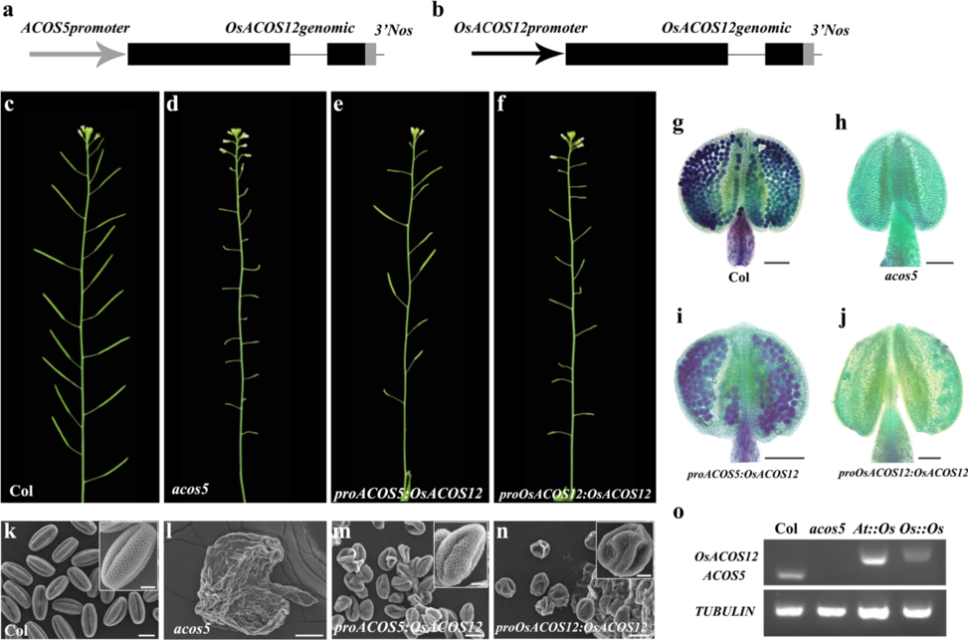
The *OsACOS12* could partially restore *acos5* fertility. **a** and **b** Structural representation of the *proACOS5*:*OsACOS12* and the *proOsACOS12*:*OsACOS12* constructs. **c**-**f** The main stem of the Col (**c**), *acos5* (**d**), *proACOS5*:*OsACOS12* with a *acos5*/*acos5* background (**e**), and *proOsACOS12*:*OsACOS12* with a *acos5*/*acos5* background (**f**) plants. **g**-**j** Alexander staining of the anthers from the Col (**g**), *acos5* (**h**), *proACOS5*:*OsACOS12* (**i**), and *proOsACOS12*:*OsACOS12* transgenic lines (**j**). Bars = 100 μm. **k**-**n** SEM examination of the dehiscent pollen grains of the wild type (**k**), *acos5* (**l**), *proACOS5*:*OsACOS12* (**m**), and *proOsACOS12*:*OsACOS12* transgenic lines (**n**). Bars = 5 μm. **o** RT-PCR analysis of *ACOS5* or *OSACOS12* expression in the flower buds of the Col, *acos5* plants and the *proACOS5*:*OsACOS12* and *proOsACOS12*:*OsACOS12* transgenic lines. *TUBULIN* was used to monitor the cDNA yield and integrity of the samples

To investigate whether the *OsACOS12* and *ACOS5* were functionally conserved, genetic complementation of the Arabidopsis *acos5* mutant (cs919318, Additional file 3: Figure S3) with the *OsACOS12* genomic sequence was performed. We generated two constructs, *proACOS5*:*OsACOS12* and *proOsACOS12*:*OsACOS12*, with *OsACOS12* driven by *ACOS5* and *OsACOS12* promoters, respectively. After the constructs were introduced into *acos5*/+ heterozygous plants (Fig. 6a-b), we obtained 12 and 14 transgenic lines with a homozygous *acos5* background for these constructs (Additional file 4: Figure S4). All 12 transgenic lines for *proACOS5*:*OsACOS12* exhibited partial fertility compared with the complete sterility of *acos5* (Fig. 6e). RT-PCR demonstrated that *OsACOS12* was highly expressed in the transgenic lines (Fig. 6o). Alexander staining showed that these transgenic plants contained mature grains that were similar to the grains of wild type plants (Fig. 6i). However, SEM analysis showed these pollen grains still had slight morphology defects (Fig. 6m). This result showed that the expression of *OsACOS12* partially rescued the fertility of the *acos5* mutant, which suggested *OsACOS12* can fulfil the function of *ACOS5* in Arabidopsis. All the transgenic lines for *proOsACOS12*:*OsACOS12* have complete male sterility (Fig. 6f). RT-PCR demonstrated that the expression of *OsACOS12* was low in these transgenic lines (Fig. 6o). Alexander staining showed that all pollen grains in the locule were aborted during the late stages of anther development (Fig. 6j). However, SEM showed that many pollen remnants could be formed in the anthers of *proOsACOS12*:*OsACOS12* transgenic plants although these pollen grains were still defective (Fig. 6n). These results suggested that the *OsACOS12* promoter was not strong enough to drive the expression of the fatty acyl-CoA synthetase gene in Arabidopsis.

## Discussion

### *OsACOS12* is an orthologue of Arabidopsis *ACOS5* for pollen exine formation and the anther wall in rice

The biosynthesis pathway of sporopollenin precursors has been identified in rice through several genetic studies. The fatty acyl reductase (DPW) converted palmitoyl-acyl carrier protein (ACP) to palmitoyl alcohol. The *WDA1* gene participated in the biosynthesis of very long chain fatty acids. CYP704B2 and CYP703A3 could catalyse the production of w-hydroxylated fatty acids with lauric, palmitic and oleic acid [22–25, 32, 33]. In addition to these catalytic reactions, the fatty acyl-CoAs were indispensable for sporopollenin monomer synthesis. In Arabidopsis, the *ACOS5* gene esterifies medium- to long-chain fatty acids to the corresponding fatty acyl-CoAs for the biosynthesis of sporopollenin [26]. In this study, we identified that *OsACOS12* is the orthologue of *ACOS5* in rice (Fig. 1). Knockout of *OsACOS12* led to the defective exine layer of the microspore and male sterility (Figs. 2 and 3). These results were consistent with the phenotype of *acos5* in Arabidopsis [26]. These results suggest that *OsACOS12* is involved in the fatty acyl-CoAs synthesis required for sporopollenin precursors. Rice anthers have obvious orbicules and reticulate anther cuticle [24]. The cuticle is essential for rice anther development because it resists abiotic and biotic pressure [34]. Orbicules have been proposed to play a role in the translocation of sporopollenin constituents. The orbicules were barely detected in the inner surface (Fig. 3), and the wax components of the *osacos12* anther were aberrant (Fig. 4). This outcome suggested that OsACOS12 was not only essential for pollen wall formation but also involved in anther cuticle lipid metabolism. The mutants of sporopollenin-related genes including *DPW*, *CYP703A3*, *CYP704B2*, *ABCG15* in rice also exhibited the defective exine and anther epicuticle formation [22, 24, 25, 32]. This result suggests that the anther cuticle and sporopollenin synthesis share a common lipid metabolism pathway in rice. However, the reticulate anther cuticle was not affected in Arabidopsis *acos5* mutants (Additional file 5: Figure S5). This result suggests there was a divergence in the lipid pathway between Arabidopsis and rice.

### OsACOS12 was expressed in the tapetum and secreted into the locule during pollen development

The tapetum cell likely synthesizes the sporopollenin precursors and subsequently transports them into the locule to be assembled on the pollen surface and form the sexine layer [5, 8, 10, 11]. The ACOS5 is an essential enzyme for sporopollenin synthesis in Arabidopsis. It transcribes components of the tapetum, meiocytes and microspores [17, 26, 35]. *OsACOS12* is an orthologue of *ACOS5*. It exhibits a similar expression pattern to *ACOS5* at the transcript level. In the complementary line, the OsACOS12-GFP signal was initially detected in the tapetal cells. At later stages, the signal occupied the free space in the locule but not in the microspores (Fig. 5). During anther development, all tapetal cellular degradation products, including proteins, entered the anther locule after it underwent the PCD process [36]. Given the secretary function of the tapetum, OsACOS12 is likely to be translated in the tapetum and secreted into the locule. Therefore, the OsACOS12 expression pattern at the protein level was different from the transcription level. This trend was also observed for *DYT1*, an essential regulator for early tapetum development. Its transcript was detected in meiocytes, the tapetum and microspores. However, DYT1 protein was only detected in tapetal cells [37]. The expression pattern of OsACOS12 was consistent with the genes involved in the biosynthesis and transportation processes of sporopollenin precursors. CYP703A2 catalyses the conversion of medium-chain saturated fatty acids to the corresponding monohydroxylated fatty acids in Arabidopsis [13]. It was expressed in the tapetum cells and secreted into the locule at the late stage of anther development [20]. Several lipid transfer proteins (LTPs: LTPC6, LTPC14, OsC6) have been noted for their secretory accumulation pattern [38, 39]. The LTPs bound or unbound to exine precursors are secreted from the tapetal cells to become exine layer constituents. The protein localization of OsACOS12 and CYP703A2 was different from MS2, which is another enzyme for sporopollenin synthesis in Arabidopsis. It was only localized in tapetal cells [19]. OsACOS12 might be secreted by the tapetum along with sporopollenin transportation.

### The sporopollenin biosynthesis pathway was conserved between rice and Arabidopsis

In Arabidopsis, approximately 9 genes were reported to be involved in sporopollenin biosynthesis and transportation [3]. The orthologues for several genes, including *CYP704B2*, *CYP703A3*, *OsPKS1*, *OsTKPR1*, *DPW* and *ABCG15*, have been identified in rice [22–25, 32]. In this study, *OsACOS12* was identified as an orthologue of *ACOS5. OsACOS12* driven by the *ACOS5* promoter was able to partially restore the fertility of the male sterile *acos5* mutant (Fig. 6e), which suggested that the functions of acyl-CoA synthetases were mainly conserved between monocot and dicot species. Previous studies showed that the rice *DPW* gene can completely rescue the sexine defects in the *ms2* mutant [32]. *PpASCL* in *Physcomitrella patens* could produce hydroxyalkyl a-pyrones, which was consistent with the results from the Arabidopsis orthologue *PKSA* [40]. It is likely that the functions of the sporopollenin biosynthesis genes were very conservative in land plants. In the transgenic line of *proACOS5*:*OsACOS12*, the expression of *OsACOS12* was comparable to that of *ACOS5* in wild type (Fig. 6o). However, the sexine layer of pollen grains in this transgenic line was still defective (Fig. 6e, m). This result suggests that the enzyme activity of OsACOS12 might be lower than that of ACOS5 in Arabidopsis or that there may be some functional divergence for fatty acid metabolism. The pollen wall patterning for a specific plant species was a conserved and elaborate process [41]. The slightly different substrates and products derived from *OsACOS12* probably led to the defective pollen surface in the transgenic line. A recent study also showed that several lipid metabolic enzymes for sporopollenin formation were conserved in tobacco and rice, while some products were different [23]. For the *ms2* mutant, *PpMS2* driven by the *MS2* promoter could not rescue its fertility. However, *DPW* driven by the *MS2* promoter could rescue its fertility with normal pollen wall formation [32, 42]. These results suggested that DPW and MS2 have a very similar function, while PpMS2 and MS2 have evolutionary divergence. In the *proOsACOS12*:*OsACOS12* line, *OsACOS12* is driven by its own promoter, and the transgenic plants exhibited a male sterile phenotype. The expression of *OsACOS12* in the *acos5* mutant was detected. However, its expression level was lower than *ACOS5* in wild type (Fig. 6o). This result suggests that the upstream regulators for sporopollenin synthesis in Arabidopsis could recognize the *OsACOS12* promoter. However, the activation efficiency was lower compared to that of the *ACOS5* promoter.

## Conclusion

In this study, we functionally identified the *OsACOS12* gene in rice, which is an orthologue of Arabidopsis *ACOS5*. Our results provided genetic evidence to suggest that OsACOS12 was involved in the lipidic metabolism for sporopollenin and cuticle synthesis in rice. The accumulation of OsACOS12 in tapetal cells and the anther locule suggested the processes of sporopollenin biosynthesis and transportation occurred synchronously. Genetic complementation assays indicated that *ACOS5* and *OsACOS12* were functionally conserved in general for pollen wall formation in rice and Arabidopsis. These findings provide new insights to illustrate fatty acyl-CoA synthetase function in the sporopollenin synthesis pathway of rice and provide a potential male sterile line for the utilization of heterosis in crops.

## Methods

### Plant materials and growth conditions

The *osacos12* mutant was obtained from Tilling technology [31]. Rice accessions, including *osacos12* and wild type Zhonghua11 (*O. sativa* ssp. Japonica), were grown in a paddy field at Shanghai Normal University (Shanghai, China). The Arabidopsis ecotype Columbia-0 and *acos5* plants were grown in a growth chamber at DATs of 22 °C under a 16 h light and 8 h dark photoperiod, unless specifically indicated.

### Characterization of the mutant phenotype

The male sterility mutant was crossed with Zhonghua11 to produce the F_1_ generation. The homozygous male sterility mutant was obtained in the F_2_ generation accompanied by the co-segregation assay. The Arabidopsis and rice florets were photographed with a digital camera (Nikon D7000) and an dissecting microscope (Olympus, SZX10). The wild type and *osacos12* mutant anthers were stained with alexander’s solution and observed with a Leica microscope (Leica, USA). For a semi-thin section, SEM and TEM observation, spikelets and anthers of wild-type and *osacos12* mutants at different stages were dissected to avoid experimental deviation. The embedding and observation procedures were performed as described by Lou et al [2].

### Complementation of the *osacos12* and *acos5* mutants

For functional complementation, a genomic DNA fragment including the *OsACOS12* coding region and 1428 bp promoter sequence were amplified from Zhonghua11 genomic DNA using gene-specific primers (Additional file 6: Table S1). The DNA fragment was subcloned into the pCAMBIA1300 binary vector using the pEASY-Uni seamless cloning and assembly kit with the BamHI restriction enzyme. The construct was introduced into *Agrobacterium tumefaciens* EHA105 and transformed into the calli induced from *osacos12* heterozygous seeds (Biorun, China). The T_1_ transgenic lines were genotyped to confirm the homozygous male sterility mutant by PCR and then verified by DNA sequence. OsACOS12-GFP florescence was detected using an LSM 5 PASCAL confocal laser scanning microscope (ZEISS, German).

For the transgenic rescue assay, the coding sequences of the 1047 bp promoter region of *ACOS5*, 1428 bp promoter region of *OsACOS12* and the genomic region of *OsACOS12* were generated by PCR amplification using primer star DNA polymerase (TaKaRa, Japan) and gene-specific primers (Additional file 6: Table S1). These sequences were subsequently cloned into the pCAMBIA1300 binary vector (CAMBIA, Australia). Constructs were transformed into fertile heterozygous Arabidopsis *acos5* plants. The transgenic lines were screened using 20 mg · L^-1^ hygromycin. Genotypic and phenotypic analysis of the segregation populations was then performed in the T_1_ generation.

### Phylogenetic analysis

The OsACOS12 protein sequence was used to search for orthologues from the plant species using the basic local alignment search tool (BLAST) at the National Center for Biotechnology Information (http://www.ncbi.nlm.nih.gov/). Multiple sequence alignments of the full-length protein sequences were performed using ClustalW and displayed using BOXSHADE (http://www.ch.embnet.org/software/ClustalW.html). The phylogenetic tree was generated by the MEGA6.0 program using the Neighbour-Joining method with default parameters with 1000 bootstrap replicates.

### RT-PCR and quantitative real-time PCR assay

Total RNA from roots, shoots, leaves, paleas and anthers at different developmental stages were extracted using a TRIzol kit (Life Technologies, USA). Subsequently, a 2 μg aliquot of total RNA was used as template for reverse transcription by AMV reverse transcriptase with a poly (dT12-18) primer (TOYOBO, Japan). Quantitative PCR analyses were performed with three repeats for each sample using SYBR Green Real-time PCR Master Mix (TOYOBO, Japan) and utilizing an ABI 7300 system (Life Technologies, USA). The quantitative PCR procedure and conditions were described previously by Lou et al [2]. The cDNA levels of the target genes were normalized to the internal standard gene *OsACTIN*. Three replicates of each sample were used for gene expression analysis. The relevant primer sequences are listed in Additional file 6: Table S1.

### In situ hybridization analyses

Fresh Zhonghua11 young panicles from different developmental stages were fixed in FAA immediately, embedded in paraffin, and sectioned at a thickness of 7 μm. A 443 bp fragment of *OsACOS12* cDNA was amplified from the wild type DNA with its specific primers (Additional file 6: Table S1). The PCR product was cloned into a pBluescript-SK vector (Stratagene) and then digested with BamHI or EcoRI to obtain the templates, respectively. These templates were transcribed in vitro by the T7 or T3 RNA polymerases to produce antisense or sense probes (Roche, USA). Tissue embedding, hybridization and signal detection were performed as described by Cai et al [21].

### Anther wax quantification

The anthers at the heading stage from wild type and *osacos12* mutants were collected for GC-MS analysis with approximately 1800 anthers per sample. Analyses of anther wax constituents and the surface area were performed according to previously described methods [24] with minor modifications. Anthers were extracted with 1 mL of chloroform for 1 min. The tetracosane (10 μg) (Sigma-Aldrich, USA) was added to extract chloroform as an internal standard. The resulting chloroform extract was transferred to a new glass vial and evaporated under a gentle stream of nitrogen. The remaining samples (100 μL) were converted with 20 μL of bis-(N,N-trimethylsilyl)-tri-fluoroacetamide (Sigma-Aldrich, USA) in 20 μL of pyridine for 40 min at 70 °C before GC-MS analysis (Agilent GC 6890, DB-1: column 30 m × 0.32 mm × 0.1 μm; on-column injection at 100 °C, oven temperature at 100 °C for 2 min, increasing at 10 °C · min^-1^ to 200 °C, 2 min at 250 °C, increasing at 10 °C · min^-1^ to 310 °C, 5 min at 310 °C and helium carrier gas at 2 mL · min^-1^). Quantification of wax compounds was accomplished with the internal standard by integrating the peak areas.

## Additional files

Additional file 1: Figure S1. Complementation of *osacos12* by *OsACOS12* genomic. a, e SEM observation for the WT (a) and C-*osacos12* (e) anthers. b, f The enlarged view of the epidermal surface of WT (b) and C-*osacos12* (f) anthers. c, g SEM observation for the WT (c) and C-*osacos12* (g) pollen grains. d, h The enlarged view of the epidermal surface of WT (d) and C-*osacos12* (h) pollen grains. Bars = 200 μm in a, e, 10 μm in b, c, f, g, and 1 μm in d, h. (TIF 3787 kb)
Additional file 2: Figure S2. TEM analysis of the tapetum in the wild type and *osacos12.* a,c. TEM images for the WT (a) and *osacos12* (c) tapetum at stage 8. b, d. TEM images for the WT (b) and *osacos12* (d) tapetum at stage 9. T, tapetum. Bars = 2 μm. (TIF 5079 kb)
Additional file 3: Figure S3. Gene structure of *ACOS5* and the T-DNA insertion. Exons are shown as black boxes. Introns, promoter, and untranslated regions are shown as lines. T-DNA was inserted in the first exon. LP, Left border primer; RP, right border primer. (TIF 94 kb)
Additional file 4: Figure S4. RT-PCR analysis of *acos5*/*acos5* background plants using the primer pair LP/RP, LB/RP and LB/LP. (TIF 406 kb)
Additional file 5: Figure S5. SEM observation of Col and *acos5* mutant anthers. a, d SEM observation for the Col (a) and *acos5* (d) anthers. b and c. An enlarged view of the epidermal surface of the Col anther. e and f. An enlarged view of the epidermal surface of the *acos5* anther. Bars = 100 μm in a, d, 10 μm in b, e and 1 μm in c, f. (TIF 2928 kb)
Additional file 6: Table S1. List of primers used in this study. (DOC 43 kb)

## Abbreviations

GC-MS: Gas chromatography–mass spectrometry
GFP: Green fluorescent protein
NCBI: National Center for Biotechnology Information
RT-PCR: Reverse transcription polymerase chain reaction
SEM: Scanning electron microscopy
TEM: Transmission electron microscopy
