# Supplementary figures and images for: OsACOS12, an orthologue of Arabidopsis acyl-CoA synthetase5, plays an important role in pollen exine formation and anther development in rice
Source: BMC Plant Biol. 2016 Nov 21;16:256. doi: 10.1186/s12870-016-0943-9 (PMC5117612; doi:10.1186/s12870-016-0943-9)

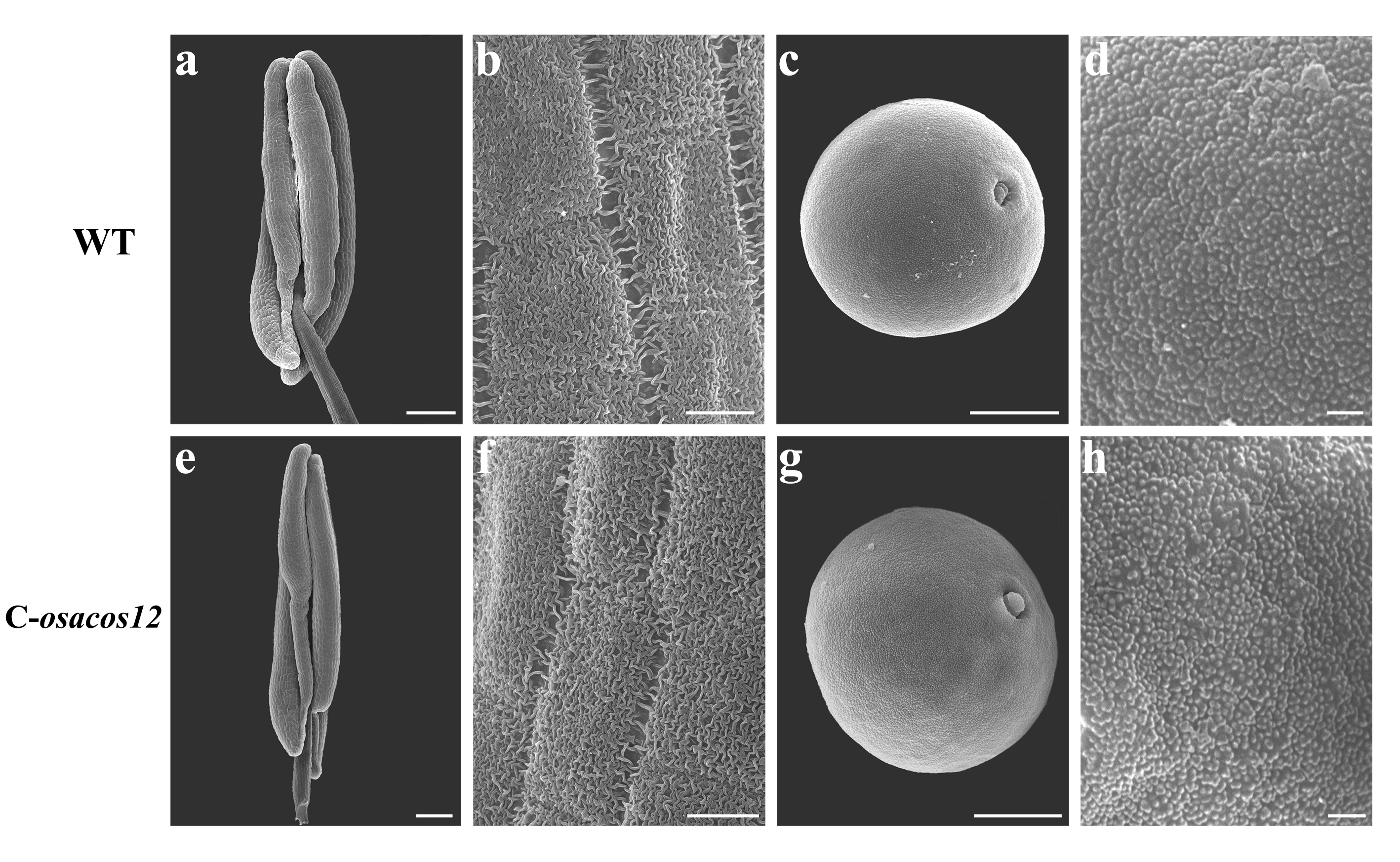

Supplement: Additional file 1: Figure S1. — Complementation of osacos12 by OsACOS12 genomic. a, e SEM observation for the WT (a) and C-osacos12 (e) anthers. b, f The enlarged view of the epidermal surface of WT (b) and C-osacos12 (f) anthers. c, g SEM observation for the WT (c) and C-osacos12 (g) pollen grains. d, h The enlarged view of the epidermal surface of WT (d) and C-osacos12 (h) pollen grains. Bars = 200 μm in a, e, 10 μm in b, c, f, g, and 1 μm in d, h. (TIF 3787 kb) [file 12870_2016_943_MOESM1_ESM.tif]

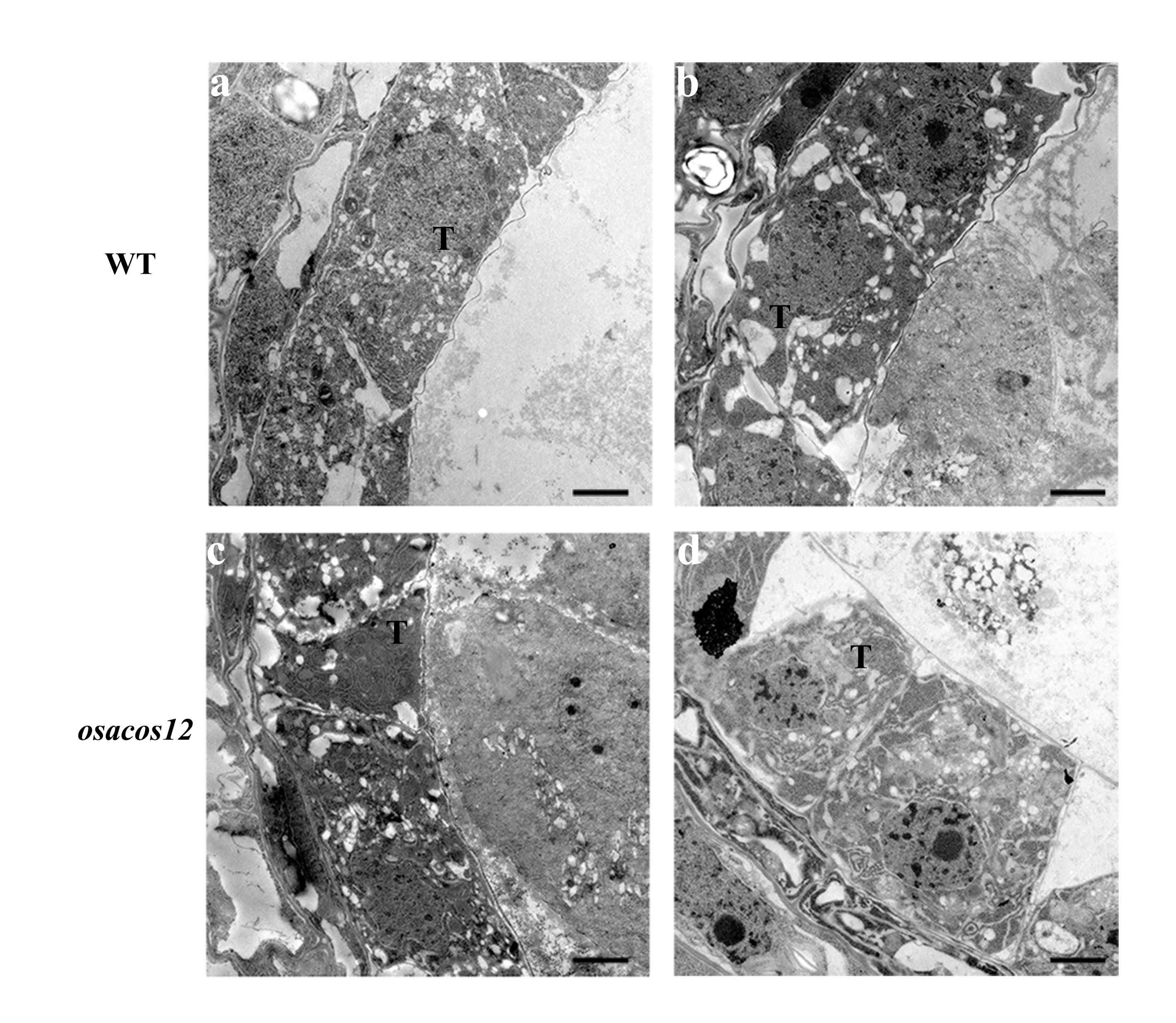

Supplement: Additional file 2: Figure S2. — TEM analysis of the tapetum in the wild type and osacos12. a,c. TEM images for the WT (a) and osacos12 (c) tapetum at stage 8. b, d. TEM images for the WT (b) and osacos12 (d) tapetum at stage 9. T, tapetum. Bars = 2 μm. (TIF 5079 kb) [file 12870_2016_943_MOESM2_ESM.tif]

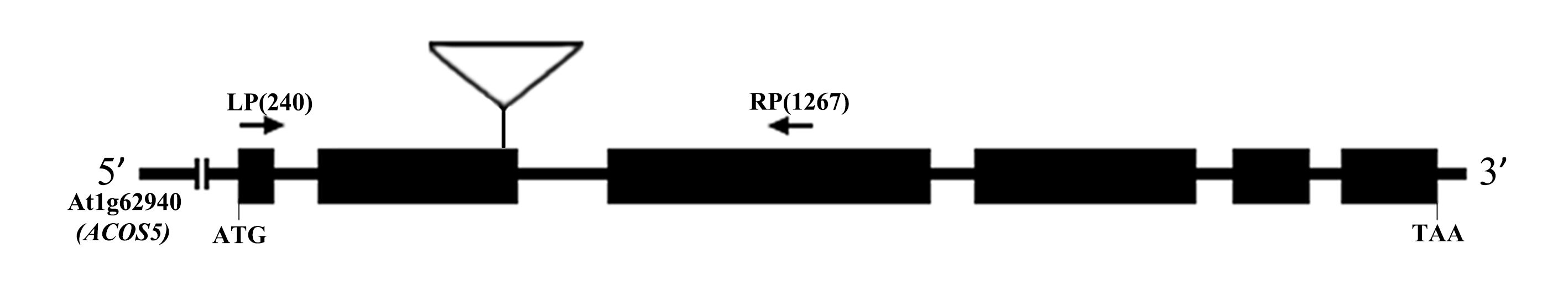

Supplement: Additional file 3: Figure S3. — Gene structure of ACOS5 and the T-DNA insertion. Exons are shown as black boxes. Introns, promoter, and untranslated regions are shown as lines. T-DNA was inserted in the first exon. LP, Left border primer; RP, right border primer. (TIF 94 kb) [file 12870_2016_943_MOESM3_ESM.tif]

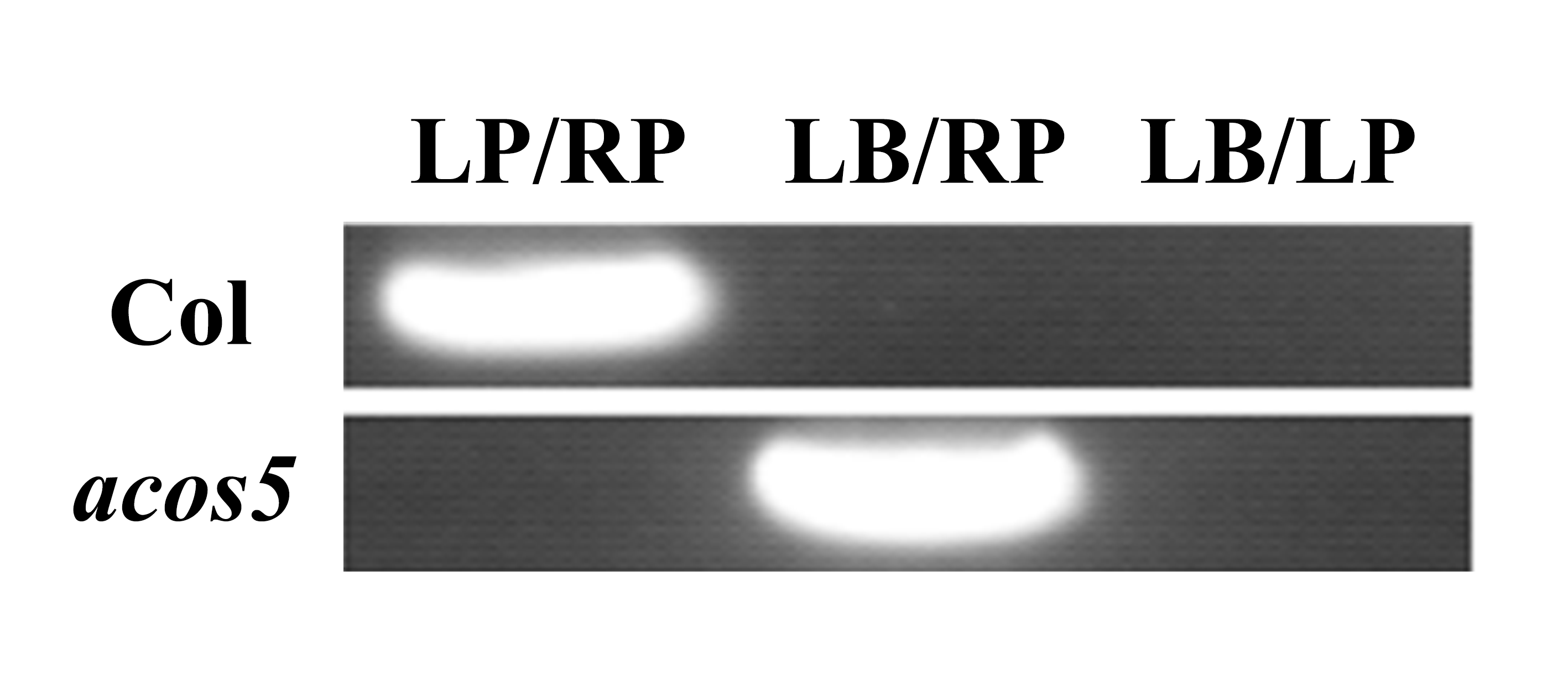

Supplement: Additional file 4: Figure S4. — RT-PCR analysis of acos5/acos5 background plants using the primer pair LP/RP, LB/RP and LB/LP. (TIF 406 kb) [file 12870_2016_943_MOESM4_ESM.tif]

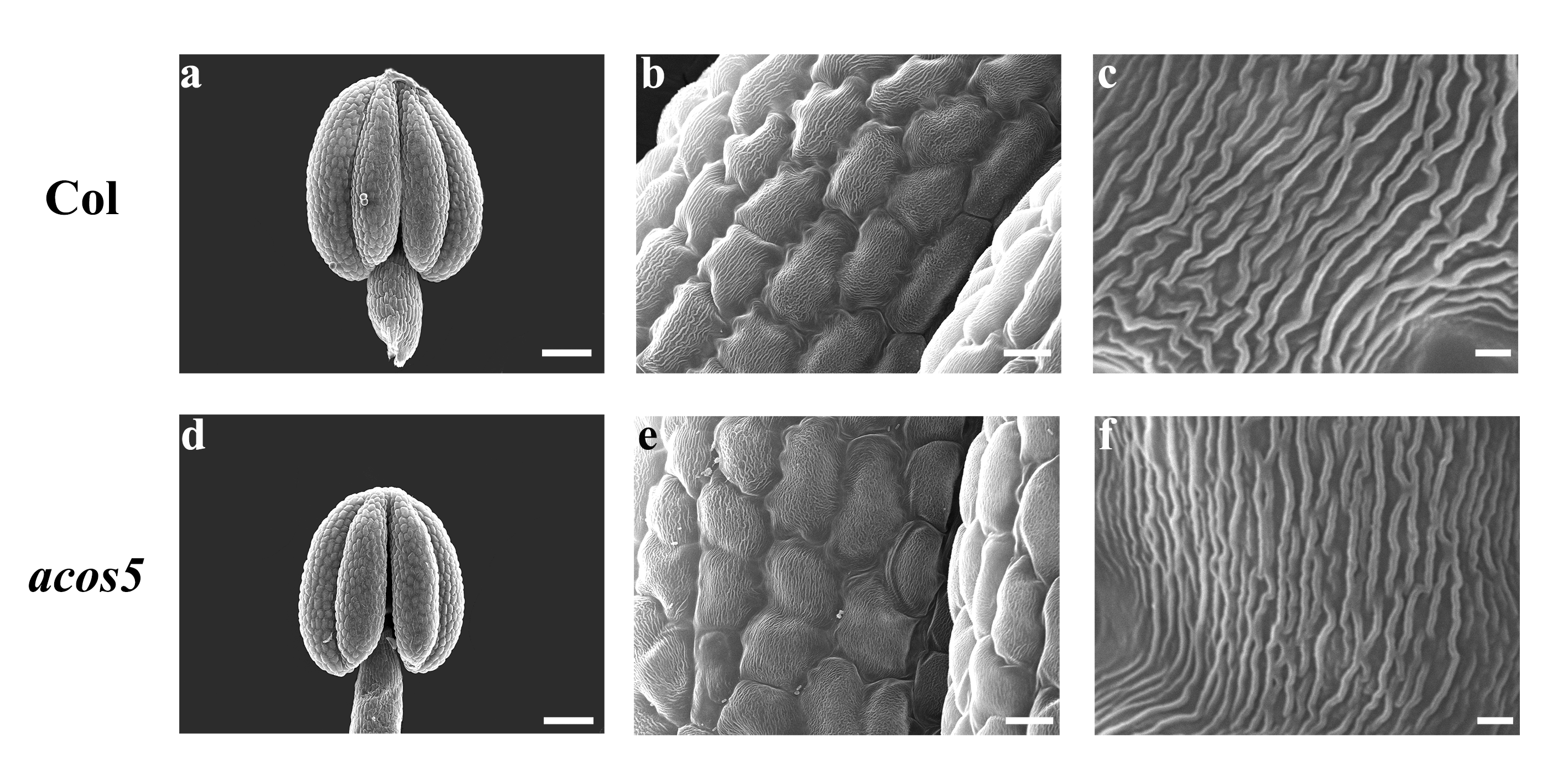

Supplement: Additional file 5: Figure S5. — SEM observation of Col and acos5 mutant anthers. a, d SEM observation for the Col (a) and acos5 (d) anthers. b and c. An enlarged view of the epidermal surface of the Col anther. e and f. An enlarged view of the epidermal surface of the acos5 anther. Bars = 100 μm in a, d, 10 μm in b, e and 1 μm in c, f. (TIF 2928 kb) [file 12870_2016_943_MOESM5_ESM.tif]
