# Supplementary material for: OsACOS12, an orthologue of Arabidopsis acyl-CoA synthetase5, plays an important role in pollen exine formation and anther development in rice
Source: BMC Plant Biol. 2016 Nov 21;16:256. doi: 10.1186/s12870-016-0943-9 (PMC5117612; doi:10.1186/s12870-016-0943-9)
Supplement: Additional file 6: Table S1. — List of primers used in this study. (DOC 43 kb) [file 12870_2016_943_MOESM6_ESM.doc]

**Supporting information**

**Table S1. List of primers used in this study..**

| Primer ID | Sequence(5’ to 3’) |
| --- | --- |
| Antisense for *in situ* F | cgggatccAGAGCGTGATGCAGGGGTACTA |
| Sense for *in situ* R | ggaattcTCCTCTTGATGAACTCGTCCCTA |
| RT-F | GGGGTACTACAAGAGGAAGGAGG |
| RT-R | CCTCTTGATGAACTCGTCCCTAA |
| QPCR-F | GAACCTGGAGGTGAAGTTCGTG |
| QPCR-R | TCAGCTCCTTGATCCTGTCCA |
| RT-Actin-F | GGCATCACACCTTCTACAACGA |
| RT-Actin-R | ATGTCCCTCACAATTTCCCG |
| QPCR-Actin-F | AATTGTGAGGGACATGAAGGAGA |
| QPCR-Actin-R | TATGAAGGAAGGCTGGAAGAGG |
| Complement *OsACOS12* genomic-F | GggatccATGGGCGACGCCGCGGTACCCGC |
| Complement *OsACOS12* genomic-R | GctgcagTCAAGCTGACGGTTTCATCCTCTTG |
| Complement *ACOS5* promoter-F | GgagctcAGTGGAAAAAGTCTGTTGTGTG |
| Complement *ACOS5* promoter-R | GggatccCGAATTGAATTTGATTTATGTG |
| Complement *OsACOS12* promoter-F | GGGGTACCCAATGAATGCTGAAGCTTATGGGG |
| Complement *OsACOS12* promoter-R | CGGGATCCGTCGCGATGCCGGTCGGC |
| Complement *osacos12* mutant-F | GCTCTAGAGATCCTTTTGAAAGCTTCTCGG |
| Complement *osacos12* mutant-R | AACTGCAGAGCTGACGGTTTCATCCTCTT |
| *ACOS5* T-DNA LP | GGCTTCGAAGGCAGTAAGAAG |
| *ACOS5* T-DNA RP | GTAGCGTTTGTAGAAGCGGTG |
| *ACOS5* T-DNA LB | TGATCCATGTAGATTTCCCGGACATGAAG |
| *osacos12* mutant-F | GTGGTGGTGATGGACCGCTT |
| *osacos12* mutant-R | AGAAGGGTGTTACCCGAAGACG |
